# Durability of treatment effects of the Sleep Position Trainer versus oral appliance therapy in positional OSA: 12-month follow-up of a randomized controlled trial

**DOI:** 10.1007/s11325-017-1568-4

**Published:** 2017-09-15

**Authors:** Maurits H. T. de Ruiter, Linda B. L. Benoist, Nico de Vries, Jan de Lange

**Affiliations:** 1 Department of Oral and Maxillofacial Surgery, Academic Medical Center, Amsterdam, The Netherlands; 2 Department of Otorhinolaryngology Head and Neck Surgery, OLVG West, Jan Tooropstraat 164 1061 AE, Amsterdam, The Netherlands; 3 Departments of Otorhinolaryngology and Head and Neck Surgery, Erasmus University Medical Center, Rotterdam, The Netherlands; 4 Department of Oral Kinesiology of the Academic Centre for Dentistry Amsterdam (ACTA), Amsterdam, The Netherlands; 5 Departments of Otolaryngology and Head and Neck Surgery, Antwerp University Hospital, Antwerp, Belgium

**Keywords:** Obstructive sleep apnea, Sleep position, Positional therapy, Oral appliance therapy, Randomized controlled trial

## Abstract

**Purpose:**

The Sleep Position Trainer (SPT) is a new option for treating patients with positional obstructive sleep apnea (POSA). This study investigated long-term efficacy, adherence, and quality of life during use of the SPT device compared with oral appliance therapy (OAT) in patients with POSA.

**Methods:**

This prospective, multicenter trial randomized patients with mild to moderate POSA (apnea-hypopnea index [AHI] 5–30/h) to SPT or OAT. Polysomnography was performed at baseline and after 3 and 12 months’ follow-up. The primary endpoint was OSA severity; adherence, quality of life, and adverse events were also assessed.

**Results:**

Ninety-nine patients were randomized and 58 completed the study (29 in each group). Median AHI in the SPT group decreased from 13.2/h at baseline to 7.1/h after 12 months (*P* < 0.001); corresponding values in the OAT group were 13.4/h and 5.0/h (*P* < 0.001), with no significant between-group difference (*P* = 1.000). Improvements throughout the study were maintained at 12 months. Long-term median adherence was also similar in the two treatment groups; the proportion of patients who used their device for ≥ 4 h for 5 days in a week was 100% in the SPT group and 97.0% in the OAT group (*P* = 0.598).

**Conclusions:**

The efficacy of SPT therapy was maintained over 12 months and was comparable to that of OAT in patients with mild to moderate POSA. Adherence was relatively high, and similar in the two groups.

**Trial registration:**

www.clinicaltrials.gov (NCT02045576).

**Electronic supplementary material:**

The online version of this article (10.1007/s11325-017-1568-4) contains supplementary material, which is available to authorized users.

## Introduction

Obstructive sleep apnea (OSA) is the most common sleep-related breathing disorder. With an overall prevalence of 9–38% in the general adult population, OSA is more common in men and increases with age [1]. Recent data from Switzerland showed that OSA was more prevalent than previously reported. The proportion of men and women with an apnea-hypopnea index (AHI) of > 5/h on polysomnography (PSG) was 84 and 61%, respectively [2]. An AHI of ≥ 5/h is required for a diagnosis of OSA, with disease severity rated as mild if the AHI is 5–15/h, moderate if the AHI is 15–30/h, and severe if the AHI is > 30/h [3].

OSA is characterized by recurrent (partial) obstruction of the upper airway, accompanied by oxygen desaturation, sleep disturbance, and sympathetic activation [4]. Consequences of OSA include excessive daytime sleepiness, reduced quality of life, and increased risk of developing cardiovascular disease. More than half of the OSA population (56%), and predominantly those with mild and moderate OSA, have position-dependent OSA (POSA) with more apneic and hypopneic events in supine position. POSA is commonly defined as more than twice as many respiratory events in the supine sleep position compared to non-supine sleep position [5–8].

Therapy for OSA generally starts with conservative treatment, consisting of lifestyle changes such as weight reduction and avoidance of alcohol, sedatives, and the supine sleeping position, when applicable. Thereafter, current options include continuous positive airway pressure (CPAP), oral appliance therapy (OAT) and pharyngeal surgery [9–11]. CPAP is the gold standard therapy for moderate to severe OSA, but adherence to CPAP is often suboptimal, necessitating exploration of other options [12]. Oral appliances (OA) are widely used in mild to moderate OSA, and are associated with clinically relevant decreases in the AHI [13], making them an increasingly attractive first-line therapy option in these patients. Vecchierini et al. reported OAT success rates of 40–70 and 78–81% in mild to moderate patients for an AHI to < 5/h and for an AHI reduction of at least 50%, respectively [13]. However, adverse events such as tooth pain, changes in tooth position resulting in a different occlusion and articulation, or temporomandibular dysfunction can limit adherence to this therapy [14, 15]. Surgery can be an option for patients who are unresponsive, noncompliant, or desire a permanent treatment for their OSA [16–18].

For POSA, alternatives include the use of specific treatments designed to avoid the supine sleeping position. However, the effectiveness of therapy with first generation devices has been limited. For example, the “tennis ball-technique” is uncomfortable for patients to use and disrupts sleep, leading to poor long-term adherence [19]. Next-generation treatment options with active feedback and auto-adapted treatment intensity to decrease discomfort and improve compliance were introduced. These include active positional therapies like supine alarm devices and neck or chest-worn vibrating devices [20, 21]. The Sleep Position Trainer (SPT) is such a chest-worn device and it showed to significantly reduce the average supine sleeping time (from 46 to 5%), the AHI to < 5/h in 48%, and an AHI reduction of at least 50% in 71% of patients with mild or moderate POSA [22]. Effectiveness and adherence were good, with an objective adherence rate (> 4 h of nightly use) of 64.4% after 6 months of treatment and improved sleep-related quality of life [23]. Additionally, short-term results have recently been published on the effectiveness of the SPT versus OAT, showing equal efficacy in reducing the median AHI in patients with mild to moderate POSA [24]. However, there are no data on the use and effect of the SPT beyond 6 months. Therefore, we aimed to study the longer-term efficacy and adherence of the SPT (the intervention) in comparison to OAT (active comparator). Hence, we hypothesized that the SPT would be more efficacious in reducing the AHI compared to OAT in patients with mild to moderate POSA. This paper investigated the durability of the previously reported short-term effects of the SPT with respect to efficacy, adherence, and quality of life, after 12 months of follow-up.

## Methods

### Participants

Participants were eligible for enrollment if they had a diagnosis of mild-to-moderate POSA (AHI of 5–30) and spent 10–90% of their total sleep time in the supine position during baseline PSG. Exclusion criteria included inadequate dentition for wearing an oral appliance, subjective snoring in the lateral position, central sleep apnea, night or rotating shift work, severe chronic heart disease, active psychiatric disease, seizure disorders, medication usage for sleeping disorders, muscular or joint injuries in the head, neck, or back area, previous OAT or SPT usage, simultaneous use of other treatment for OSA, reversible morphological upper airway abnormalities (e.g., enlarged tonsils), pregnancy, and coexisting non-respiratory sleep disorders (e.g., insomnia, periodic limb movement disorder, narcolepsy) that would compromise functional sleep assessment. All participants underwent medical and dental consultations, and a baseline PSG prior to the start of the study.

### Study design and oversight

The study was designed as a multicenter, prospective randomized controlled trial. Patients were recruited and followed at the departments of Otolaryngology and Clinical Neurophysiology at OLVG West Hospital, Amsterdam, and at the department of oral and maxillofacial surgery at the Academic Medical Center, Amsterdam. The institutional Medical Ethics Committee of the OLVG West Hospital, Amsterdam, and the Academic Medical Center Amsterdam approved the protocol. The randomization sequence was generated by an independent clinical research unit using ALEA software with a 1:1 allocation using maximum random block sizes of 6 and stratification for smoking and body mass index (BMI). Independent monitors verified the source data and documentation.

### Study treatments

**Fig. 1:**
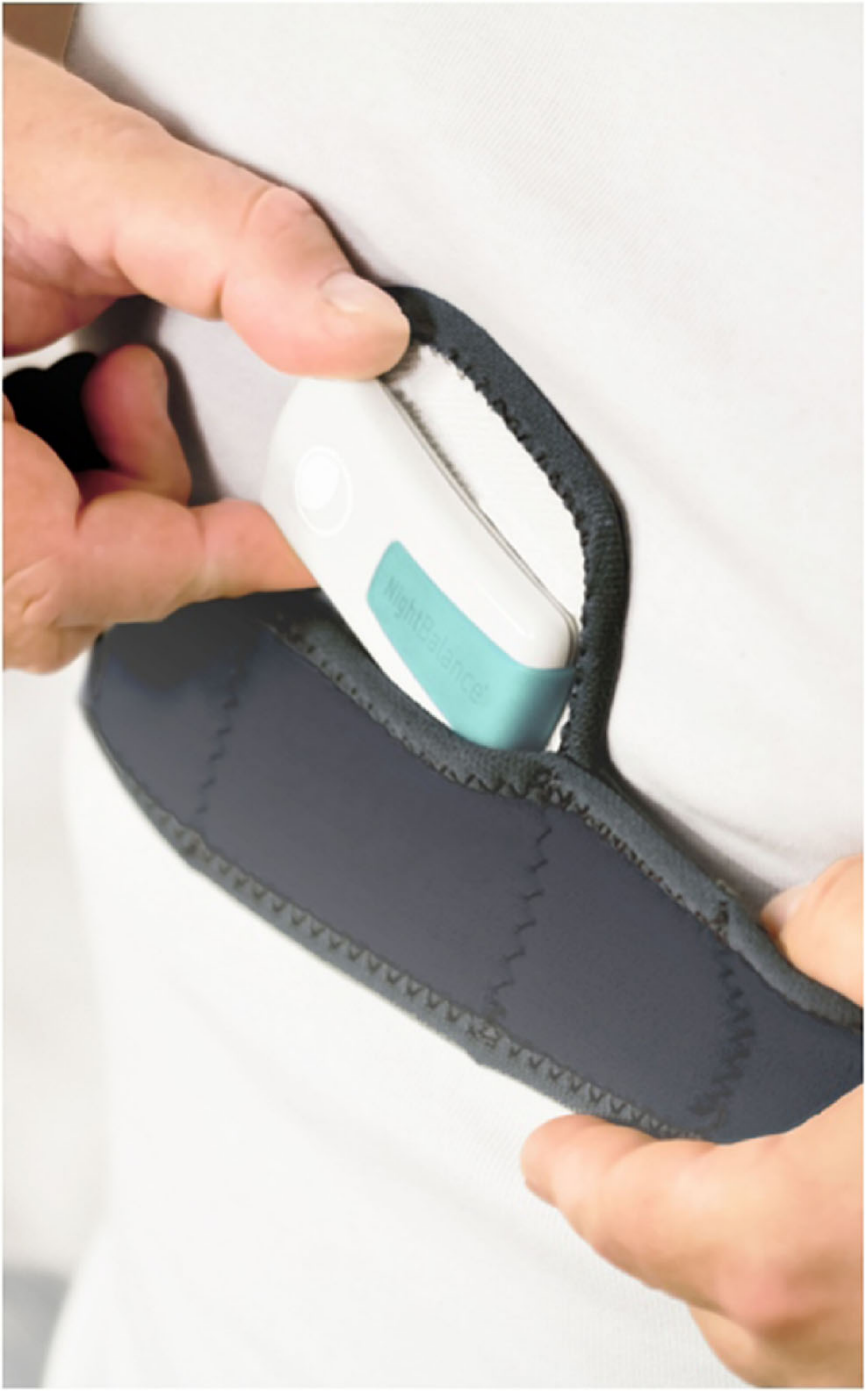
Sleep Position Trainer

The sleep position trainer (SPT-DEV-PX-11.08; NightBalance) consists of a small lightweight device (72 × 35 × 10 mm; 25 g) worn across the chest using a neoprene strap (Fig. 1) [22]. The SPT vibrates when a supine position is detected to prompt a change in body position. Data storage on the device allows for objective measurement of adherence to the therapy. Further details on functionality of the SPT are described elsewhere [24].

**Fig. 2:**
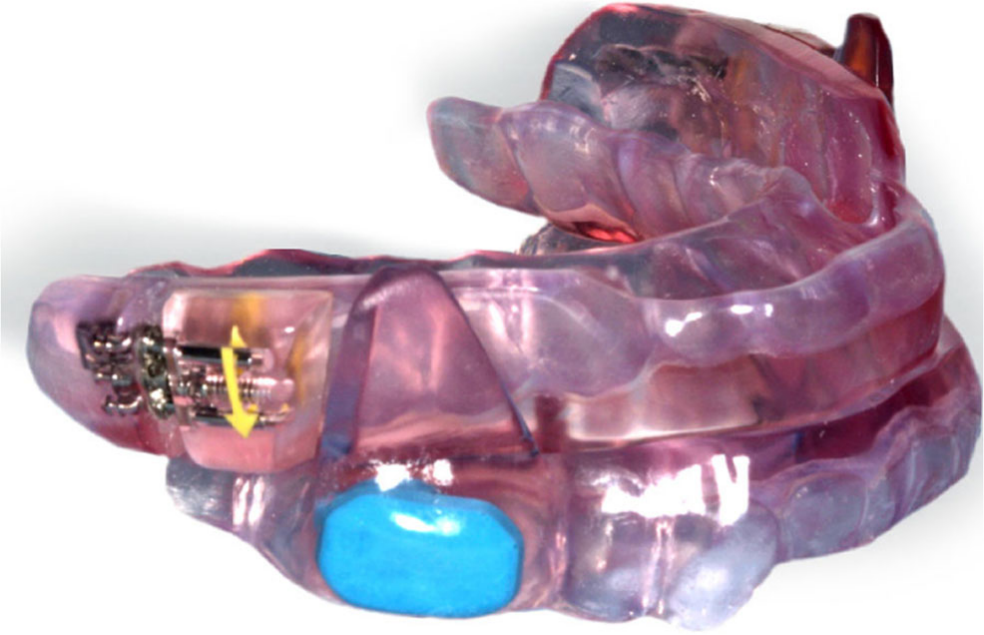
Oral appliance therapy, including a blue chip for measuring adherence

As active comparator, the OA was a custom-made duo-bloc device (SomnoDent flex; SomnoMed) (Fig. 2). After adequate assessment of the central relation and maximum protrusion, the OA was set at 60% of maximum protrusion at baseline. The OA was adjusted individually and advancement was titrated using a standard protocol by the dentist, which was described in greater detail elsewhere [24]. Objective adherence was measured using a temperature-sensitive microsensor with on-chip integrated read-out electronics (Theramon, Handels- und Entwicklungsgeselschaft, Handelsagentur Gschladt, Hargelsberg, Austria) with a sampling rate of one measurement every 15 min. A recorded temperature of > 30 °C indicated that the OA was worn.

### Outcome measures

The primary outcome was the change in OSA severity after 12 months compared with baseline. OSA severity was determined based on the AHI and the oxygen desaturation index (ODI; the number of times per hour of sleep that the blood oxygen level drops by ≥ 4% from baseline, according to the prevailing definition at that time). These parameters were determined from overnight PSG (Embla A10, Broomfield, CO, USA) which records electroencephalogram (EEG) (FP2-C4/C4-O2), electro-oculogram (EOG), electrocardiogram (ECG), and submental and anterior tibial electromyogram (EMG). Nasal airflow was measured by a nasal pressure cannula and blood oxygen saturation by finger pulse oximetry. Straps containing piezoelectric transducers recorded thoracoabdominal motion, and a position sensor (Sleepsense, St Charles, IL, USA) attached to the midline of the abdominal wall was used to differentiate between supine, prone, right lateral, left lateral, and upright positions. Recordings were manually scored by an independent core laboratory using American Academy of Sleep Medicine (AASM) 2012 scoring criteria [25]. Secondary outcomes included additional polysomnographic variables, percentage of time spent sleeping in the supine position, AHI in the supine and non-supine positions, and sleep efficiency. Self-reported daytime sleepiness was assessed using the Epworth Sleepiness Scale (ESS; score range 0–24, score ≥ 10 indicates excessive daytime sleepiness). Disease-specific quality of life was assessed with the Functional Outcomes of Sleep Questionnaire (FOSQ-30; score range 5–20, higher scores indicate better functioning). Adherence was defined as device (SPT or OAT) usage for ≥ 4 h/night at least 5 days per week.

### Follow-up

This paper aimed at testing the durability of treatment effect after 12-month follow-up. Throughout the follow-up, patients underwent repeat PSG at 3 and 12 months, while using the SPT or OAT. Patients completed the ESS and FOSQ-30 at baseline and after 3 and 12 months of therapy. Objective adherence and medical evaluation (including heart rate and blood pressure measured twice seated by two independent physicians with an interval of 3 min) were also assessed at 3 and 12 months.

### Adverse events

Adverse events were reported in accordance with the International Conference of Harmonization ICH E2A guidelines (Good Clinical Practice) by the principal investigators and evaluated by independent data monitors [26].

### Statistical analysis

Power analysis resulted in a minimum sample size of 36 participants per study arm (to reach a power of 80%). In order to allow for dropout, the recruitment target was inflated to 49 per group. The level for statistical significance was set at *α* = 0.05.

The statistical programming and analysis was performed using IBM SPSS statistics version 24 (IBM Corp., Armonk. NY, USA). Due to the proportion of missing data at 12 months, analyses were primarily conducted on a per-protocol (PP) basis. Additionally, illustrative worst-case and best-case intention-to-treat (ITT) analyses were performed through imputing missing data through the Last-Observation-Carried-Forward method.

Variables were summarized using descriptive statistics: mean value with standard deviation for continuous symmetric variables, median and interquartile range for continuous skewed variables, and frequency with percentage for categorical variables. For the primary outcome, repeated measures ANOVA was performed to test for differences over time. Thereafter, within-subject comparisons (patient progression over time; paired) between continuous variables at baseline and follow-up (3 and 12 months) and between the 3- and 12-month follow-ups were made using the paired *t* test (non-skewed data) or the Wilcoxon signed rank test (skewed data). Between-group difference tests (deltas baseline vs. follow-up) were performed using an independent *t* test (non-skewed data) or Mann-Whitney *U* test (skewed data). Both the between-group and within-subject analyses were adjusted for multiple comparisons using the Bonferroni correction.

## Results

**Fig. 3:**
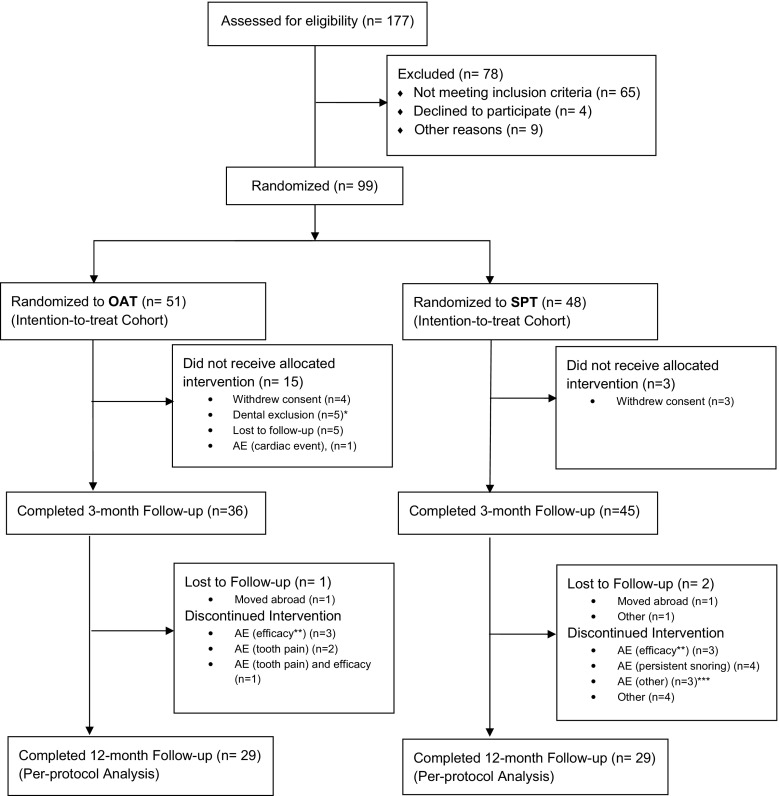
Flow of patients through the study. AE, adverse event; OAT, oral appliance therapy; SPT, Sleep Position Trainer. *Although insufficient dental status was an exclusion criterion, a dentist checked this through regular physical examination. Some dental problems were only visualized after the orthopantomography was made. ** Efficacy: persistent apneas/AHI. *** Adverse events; one related events (joint problems due to wearing SPT), two non-related events (one patient had nasal problems and was not motivated to continue and one patient had broken ribs due to an accident and did not want to continue)

A total of 177 patients with mild to moderate POSA were screened for eligibility (70.7% male, age 48.3 ± 10.1 years; BMI 27.6 ± 3.8 kg/m^2^). Of these, 99 patients met all eligibility criteria and were randomized to OAT (*n* = 51) or SPT (*n* = 48) (Fig. 3). Baseline characteristics for these patients are shown in Table 1 with comparison of the characteristics between “completers” and “dropouts.” There was only a statistically significant difference in blood pressure between the OAT and SPT groups at baseline (Table 1). The total number of patients receiving allocated treatment with OAT and SPT, and completing 3 months’ follow-up was 36 and 45, respectively. Over the remaining 9 months of follow-up, an additional seven patients withdrew in the OAT group (one lost to follow-up and six discontinued treatment due to adverse events [*n* = 2], lack of efficacy [*n* = 3], or both adverse event and efficacy [*n* = 1]) (Fig. 3). In the SPT group, 2 patients were lost to follow-up and 14 discontinued treatment (lack of efficacy [*n* = 3], persistent snoring [*n* = 4], adverse events [*n* = 3; 2 not related to SPT], or other non-related reasons [*n* = 4]) (Fig. 3). A total of 58 patients were eligible for per-protocol analysis after 12 months (Fig. 3).

**Table 1:** Baseline characteristics in all randomized patients (*n* = 99) and comparison between “completers” and “dropouts”

	SPT			OAT			*P* value^a^	*P* value^b^
	Total (*n* = 48)	Completers (*n* = 29)	Dropouts (*n* = 19)	Total (*n* = 51)	Completers (*n* = 29)	Dropouts (*n* = 22)		
Male, *n* (%)	34 (70.8)	19 (65.5)	18 (62.1)	36 (70.6)	15 (78.9)	18 (81.8)	0.979^c^	0.340^c^
Age, years	47.3 ± 10.1	49.5 ± 9.4	49.5 ± 8.5	49.2 ± 10.2	43.8 ± 10.3	48.9 ± 12.3	0.347^d^	0.209^f^
Body mass index, kg/m^2^	27.5 ± 2.9	27.7 ± 2.8	28.3 ± 3.6	27.7 ± 4.5	27.1 ± 2.9	26.8 ± 5.5	0.797^d^	0.501^f^
Height, cm	177.0 ± 10.2	176.7 ± 11.2	172.7 ± 12.1	174.4 ± 11.9	177.4 ± 8.8	176.5 ± 11.6	0.247^d^	0.422^f^
Weight, kg	86.2 ± 13.2	86.9 ± 14.9	84.2 ± 11.4	83.9 ± 14.2	85.1 ± 10.3	83.5 ± 17.4	0.401^d^	0.820^f^
Neck circumference, cm	38.0 ± 3.6	37.9 ± 3.8	37.2 ± 3.1	37.7 ± 3.2	38.3 ± 3.4	38.3 ± 3.2	0.624^d^	0.655^f^
Smokers, *n* (%)	11 (22.9)	5 (17.2)	7 (24.1)	12 (23.5)	6 (31.6)	5 (22.7)	0.942^c^	0.719^c^
Alcohol intake, *n* (%)								
≤ 2 drinks/day	45 (93.7)	26 (89.7)	27 (93.1)	48 (94.1)	19 (100.0)	21 (95.5)	0.446^c^	0.688^c^
> 2 drinks/day	3 (6.3)	3 (10.3)	2 (6.9)	3 (5.9)	0 (0.0)	1 (4.5)		
Blood pressure, mmHg								
Systolic	135.0 (125.0–150.0)	130.0 (125.0–150.0)	130.0 (120.0–141.0)	130.0 (120.0–140.0)	140.0 (122.0–155.0)	129.0 (120.0–136.3)	0.032^e^	0.162^f^
Diastolic	90.0 (80.0–97.5)	80.0 (90.0–95.0)	85.0 (80.0–90.0)	85.0 (80.0–90.0)	90.0 (80.0–100.0)	82.5 (79.5–90.0)	0.033^e^	0.141^f^
Heart rate, beats/min	69.0 (64.0–78.0)	68.0 (63.0–78.0)	72.0 (62.0–78.0)	72.0 (66.0–80.0)	74.0 (64.0–80.0)	71.0 (67.0–82.3)	0.530^e^	0.292^f^
AHI, /h	13.0 (9.7–18.5)	13.2 (10.2–19.0)	13.4 (8.7–16.9)	11.7 (9.0–16.2)	12.1 (7.0–17.2)	10.3 (9.0–13.3)	0.318^e^	0.222^f^
Supine AHI, /h	27.0 (18.7–43.1)	28.5 (18.9–46.2)	25.8 (15.8–45.1)	25.8 (17.4–35.0)	26.0 (11.6–36.8)	26.1 (18.8–34.2)	0.687^e^	0.491^f^
Non-supine AHI, /h	3.5 (1.6–5.7)	4.1 (2.4–5.8)	3.8 (0.8–5.7)	3.1 (1.0–5.0)	2.4 (0.9–5.7)	2.6 (1.2–3.7)	0.361^e^	0.057^f^
Supine sleep time, %	44.5 (30.0–55.5)	41.0 (30.0–54.0)	35.0 (25.0–61.0)	39.0 (26.0–54.0)	47.0 (25.0–57.0)	42.5 (27.5–47.5)	0.575^e^	0.901^f^
ODI, /h	10.5 (7.0–15.8)	9.0 (7.0–15.5)	10.0 (6.5–14.0)	9.0 (6.0–14.0)	13.0 (7.0–16.0)	8.0 (5.5–11.8)	0.137^e^	0.218^f^
AI, /h	9.0 (5.0–15.0)	11.0 (5.5–15.5)	8.0 (3.5–12.5)	8.0 (4.0–12.0)	7.0 (3.0–11.0)	7.0 (3.8–11.3)	0.183^e^	0.310^f^
Sleep efficiency, %	92.0 (84.5–95.0)	92.0 (84.0–95.5)	91.0 (85.5–95.0)	92.0 (86.0–95.0)	92.0 (89.0–94.0)	93.0 (87.8–96.3)	0.820^e^	0.811^f^
Mean oxygen saturation, %	95.0 (94.0–96.8)	95.0 (94.5–96.0)	95.0 (94.0–96.0)	95.0 (94.0–96.0)	95.0 (94.0–97.0)	95.5 (94.0–96.3)	0.451^e^	0.575^f^
ESS score	8.1 ± 5.2 (*n* = 42)	8.9 ± 5.7	6.9 ± 4.4	8.7 ± 5.6 (*n* = 45)	7.1 ± 4.3	10.7 ± 6.3	0.625^e^	0.073^f^
FOSQ score	19.0 (17.3–19.7) (*n* = 33)	18.9 (16.8–19.5)	19.3 (16.9–19.8)	18.4 (16.2–19.7 (*n* = 40)	19.4 (18.8–19.7)	18.3 (16.2–19.4)	0.646^e^	0.864^f^

Values are mean ± standard deviation, median (interquartile range), or number of patients (%)

*AHI* apnea-hypopnea index, *AI* apnea index, *ESS* Epworth Sleepiness Scale score, *FOSQ* Functional Outcomes of Sleep Questionnaire, *OAT* oral appliance therapy, *ODI* oxygen desaturation index, *SPT* Sleep Position Trainer

^a^Comparing total group scores between SPT and MAD (two groups)

^b^Comparing completers’ and dropouts’ scores between SPT and MAD (four groups)

^c^Pearson chi-square test

^d^Independent *T* test

^e^Mann-Whitney test

^f^One-way ANOVA

### Primary outcome

PP analysis showed that the AHI and ODI were significantly reduced compared with baseline at both the 3- and 12-month follow-up visits for both treatment groups, with no significant between-group differences (Table 2). The absolute reductions in AHI and ODI at 3 months were maintained at 12 months in both groups (Table 2). ITT analysis for the primary outcome is provided in Table S1. The AHI reduced for more than 50% in 48.3 and 51.7% of SPT patients after 3 and 12 months, respectively. For the OAT group, this reduction was found in 48.3% patients after 3 months and 55.2% patients after 12 months of follow-up. The outcomes were not statistically different between the two treatment groups (*P* = 1.000 at 3 months and *P* = 0.792 at 12 months). Alternatively, a reduction of the AHI under 5/h for the 3- and 12-month follow-up was found in 34.5 and 41.4% of SPT patients and 41.4 and 51.7% of OAT patients, respectively. These outcomes were also not significantly different between the groups (*P* = 0.5888 at 3 months and *P* = 0.430 at 12 months).

**Table 2:** Primary and secondary outcome variables (per-protocol analysis)

	SPT (*n* = 29)			OAT (*n* = 29)		
	Baseline	3 months	12 months	Baseline	3 months	12 months
Primary outcome						
Total AHI, /h	13.2 (10.2, 19.0)	*6.8 (4.* *1, 11.5)* ^a^	*7.1 (4.0, 10.0)* ^a^	13.4 (8.7, 16.9)	*5.9 (3.8, 9.6)* ^a^	*5.0 (3.9, 8.9)* ^a^
ODI, /h	9.0 (7.0, 15.5)	*5.0 (4.0, 8.0)* ^a^	*6.0 (3.0, 8.0)* ^a^	10.0 (6.5, 14.0)	*7.0 (4.0, 9.0)* ^b^	7.0 (3.0, 10.5)
Secondary outcomes						
Supine AHI, /h	28.5 (18.9, 46.2)	*12.4 (0.0, 34.3)* ^c^	*10.0 (0.0, 20.2)* ^a^	25.8 (15.8, 45.1)	*14.3 (5.6, 26.8)* ^a^	*10.3 (5.5, 18.3)* ^a^
Non-supine AHI, /h	4.1 (2.4, 5.8)	4.3 (2.0, 7.2)	4.5 (2.6, 7.8)	3.8 (0.8, 5.7)	1.6 (0.6, 4.0)	1.8 (0.5, 4.5)
Supine sleep, %	41.6 ± 17.0	*13.3 ± 12.9* ^a, d^	*12.7 ± 13.6* ^a, d^	42.3 ± 21.4	41.4 ± 26.4	43.9 ± 23.7
AI, /h	11.0 (5.5, 15.5)	*4.0 (1.0, 8.5)* ^a^	*3.0 (2.0, 6.0)* ^a^	8.0 (3.5, 12.5)	*3.0 (1.0, 6.5)* ^a^	*2.0 (1.0, 5.0)* ^a^
Sleep efficiency, %	92.0 (84.0, 95.5)	92.0 (89.5, 96.0)	91.0 (84.5, 95.5)	91.0 (85.5, 95.0)	91.0 (84.5, 94.0)	93.0 (86.0, 95.0)
Average SpO_2_, %	95.0 (94.5, 96.0)	96.0 (95.0, 97.0)	96.0 (94.5, 97.0)	95.0 (94.0, 96.0)	95.0 (93.5, 96.0)	94.0 (94.0, 96.5)
SBP, mmHg	130.0 (125.0, 150.0)	*125.0 (120.0, 135.0)* ^b, e^	130.0 (120.0, 142.5)	130.0 (120.0, 141.0)	125.0 (122.5, 137.5)	125.0 (120.0, 139.0)
DBP, mmHg	90.0 (80.0, 95.0)	*80.0 (75.0, 90.0)* ^a, f^	*80.0 (80.0, 90.0)* ^b^	85.0 (80.0, 90.0)	85.0 (80.0, 90.0)	80.0 (80.0, 85.0)
Heart rate, bpm	68.0 (63.0, 78.0)	70.0 (66.5, 80.0)	72.0 (69.0, 80.0)	72.0 (62.0, 78.0)	70.0 (63.0, 80.0)	74.0 (67.0, 80.0)
ESS score^g^	9.0 (3.5, 12.8)	7.0 (5.0, 10.0)	7.0 (3.5, 10.0)	6.0 (4.0, 10.8)	4.5 (3.0, 7.0)	4.0 (2.0, 8.0)
FOSQ score^h^	18.9 (16.8, 19.5)	18.9 (17.0, 19.9)	19.0 (18.2, 19.7)	19.3 (16.9, 19.8)	18.5 (16.1, 19.6)	17.7 (16.9, 19.9)

Values are mean ± standard deviation or median (interquartile range). *P* values are adjusted for multiple comparisons by a Bonferroni correction

*AHI* apnea-hypopnea index, *AI* apnea index, *bpm* beats/min, *DBP* diastolic blood pressure, *ESS* Epworth Sleepiness Scale, *FOSQ* Functional Outcomes of Sleep Questionnaire, *OAT* oral appliance therapy, *ODI* oxygen desaturation index, *SBP* systolic blood pressure, *SpO*_*2*_ oxygen saturation, *SPT* Sleep Position Trainer

^a^ *P* < 0.001 vs baseline (Wilcoxin signed rank test)

^b^ *P* < 0.01 vs. baseline (Wilcoxin signed rank test)

^c^ *P* < 0.05 vs. baseline (Wilcoxin signed rank test)

^d^ *P* < 0.001 vs. OAT (Mann-Whitney *U* test)

^e^ *P* < 0.05 vs. OAT (Mann-Whitney *U* test)

^f^ *P* < 0.01 vs. OAT (Mann-Whitney *U* test)

^g^Data available in 24, 27, 21 patients in the SPT group and 24, 24, 25 patients in the OAT group for baseline, 3 months, and 12 months, respectively

^h^Data available in 19, 19, 12 patients in the SPT group and 20, 18, 18 patients in the OAT group for baseline, 3 months, and 12 months, respectively

**Fig. 4:**
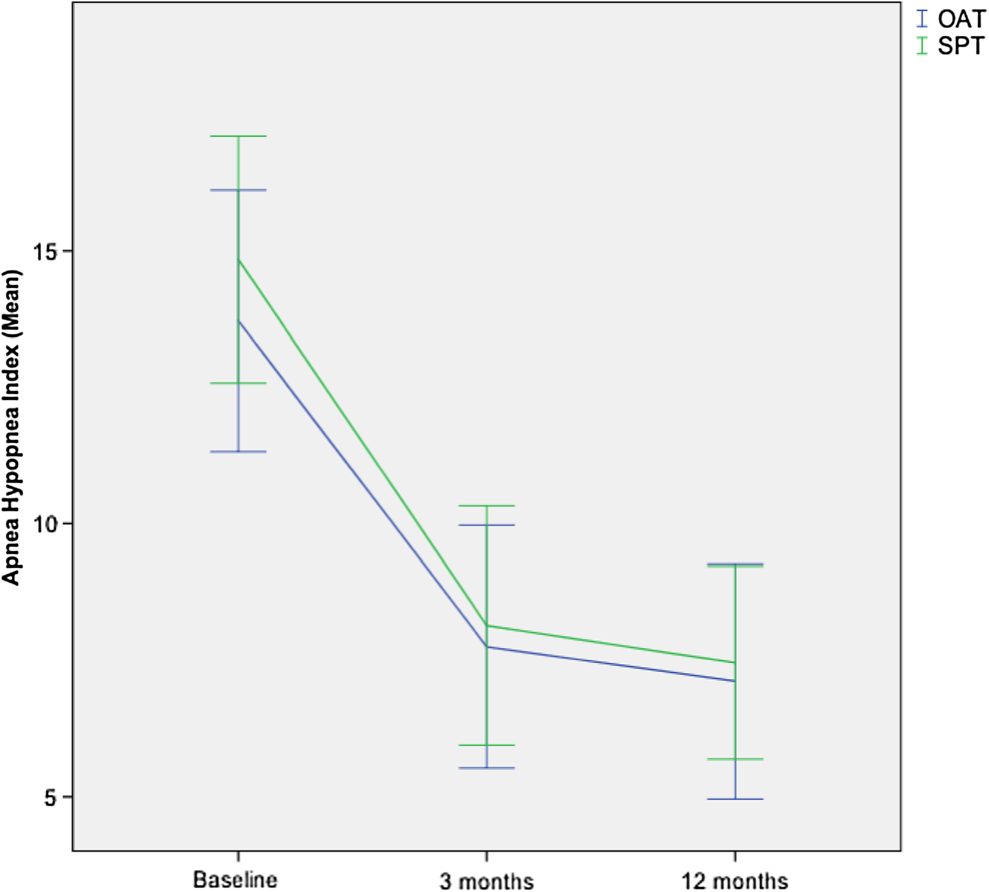
Durability of effects on the apnea-hypopnea index (AHI) over time in the Sleep Position Trainer (SPT) and oral appliance therapy (OAT) groups (ANOVA repeated measures)

Durability of the treatment effect of both the SPT and OAT groups was good (Fig. 4). There was a statistically significant effect of time on the AHI under treatment (*F*(2, 56) = 65.97, *P* < 0.001), with no significant between-group difference (*P* = 0.592). For the reduction in AHI, stratification by OSA severity at baseline (mild [*n* = 34) vs. moderate [*n* = 24]) was performed (*F*(2, 54) = 102.39, *P* < 0.001). However, no severity-related difference in reduction of AHI was observed between the treatment arms (*P* = 0.200).

### Secondary outcomes

#### Polysomnographic indices

Treatment with SPT was associated with a significant decrease in supine sleeping time (*P* < 0.001 vs. baseline after 3 and 12 months), but supine sleeping time was unchanged from baseline in the OAT group (between-group difference, *P* < 0.001) (Table 2). Supine AHI decreased to a similar extent in the two groups (Table 2). Sleep efficiency remained stable over the 12-month follow-up, and no significant changes in cardiovascular parameters were observed between the two treatment groups (Table 2).

#### Adherence

Device usage and adherence were similar in the SPT and OAT groups throughout the 12-month follow-up (Table 3 and S2). The average usage per night was 5.2/h for SPT and 5.0/h for OAT (*P* = 0.743). Median adherence per patient (≥ 4 h for 5 days/week) was 100% in the SPT group and 97.0% in the OAT group (*P* = 0.598).

**Table 3:** Objective adherence and device usage (per-protocol analysis)

	SPT (*n* = 29)	OAT (*n* = 28)	*P* value
Total nights	365.0 (362.5–365.0)	356.5 (275.8–373.5)	0.805^a^
Total nights with adherence > 4 h	237.6 ± 96.3	239.9 ± 96.1	0.930^b^
Average hours of use per night	5.2 ± 2.2	5.0 ± 2.0	0.743^b^
Adherence > 4 h on 7 days in a week, % patients	82.0 (47.0–90.5)	79.8 (59.7–97.4)	0.314^a^
Adherence > 4 h on 5 days in a week, % patients	100.0 (65.5–100.0)	97.0 (79.9–100.0)	0.598^a^

Values are mean ± standard deviation or median (interquartile range). One patient missing data in OAT group

*OAT* oral appliance therapy, *SPT* Sleep Position Trainer

^a^Mann-Whitney test

^b^Independent *T* test

#### Subjective daytime sleepiness and sleep-related quality of life

Complete 12-month data from the ESS questionnaire were available for 21/29 (72%) and 25/29 (86%) patients in the SPT and OAT groups, respectively; corresponding values for completion of the FOSQ were 12/29 (41%) and 13/29 (45%). No significant changes in the ESS score and FOSQ score were identified in either treatment group (Table 2).

#### Adverse events

A total of 114 device-related adverse events (AE) were reported by 48 patients (82.8%) overall, 20 (69.0%) in the SPT group, and 28 (96.6%) in the OAT group (Table 4). Overall, the most common adverse events in both groups were persistent snoring and persistent tiredness. A similar degree of persistent snoring was reported for SPT and OAT; by 14 and 15 patients, respectively. However, for an additional four SPT patients, persistent snoring was a reason for dropping out of the study (Fig. 3). The most common SPT-specific adverse events were being woken by the vibration and no reaction to the vibration. In the OAT group, the most common device-specific events were tooth pain, temporomandibular dysfunction, and open bite. In patients treated with the SPT, no events necessitated a temporary discontinuation of device use. For the OAT group, study treatment was temporarily discontinued as a result of six events in a total of five patients (17.2% of patients). The number of device-specific adverse events (vs. non-specific events) was higher in the OAT group (44 vs. 8 in the SPT group; *P* < 0.001). No statistical difference was found in the duration (in days) of adverse events (*P* = 0.830) between the groups.

**Table 4:** Adverse events

	Total	SPT	OAT
Number of subjects (%)	58 (100)	29 (100)	29 (100)
Reporting at least 1 AE (%)	48 (82.8)	20 (69.0)	28 (96.6)
Frequency of events (%)	114 (100)	37 (100)	77 (100)
Persistent snoring (%)	29 (25.4)	14 (37.8)	15 (19.5)
Persistent tiredness (%)	21 (18.4)	7 (18.9)	14 (18.2)
Persistent apneas (%)	1 (0.9)	1 (2.7)	0 (0.0)
Comfort problems (%)	7 (6.1)	5 (13.5)	2 (2.6)
Other (%)	4 (3.5)	2 (5.4)	2 (2.6)
OAT			
Tooth pain (%)	21 (18.4)		21 (27.3)
TMD (%)	9 (7.9)		9 (11.7)
Open bite (%)	7 (6.1)		7 (9.1)
Dry mouth (%)	4 (3.5)		4 (5.2)
Hypersalivation (%)	1 (0.9)		1 (1.3)
Dental fracture (%)	1 (0.9)		1 (1.3)
Oral lesions (%)	1 (1.3)		1 (1.3)
SPT			
Woken up by vibration (%)	4 (3.5)	4 (10.8)	
No reaction to vibration (%)	4 (3.5)	4 (10.8)	

*AE* adverse event, *OAT* oral appliance therapy, *SPT* Sleep Position Trainer, *TMD* temporomandibular dysfunction

## Discussion

The results of this study in patients with POSA showed that the beneficial effects of both the SPT and OAT observed at 3 months’ persisted through 12 months of device use. The SPT improved sleep apnea to a similar extent as OAT and was associated with high adherence rates. This is the first long-term, randomized controlled trial comparing positional therapy using the SPT with OAT for the treatment of POSA.

These findings are consistent with previous short-term data on the SPT [22, 27] and confirm that benefits are maintained over a longer-term follow-up. It is important to assess OSA therapies over longer periods of time because many, including CPAP, show reduced adherence over time. When adherence is defined as device usage for > 4 h/night, 46–83% of CPAP users are nonadherent [12]. Objective data on use of OAT have shown that 83% of patients used the device regularly [28]. Adherence rates for OAT in our study were similar, and SPT device usage was also of a similar magnitude. In this study, SPT had similar long-term efficacy to OAT and was associated with consistently high levels of adherence over 12 months’ follow-up, highlighting the potential clinical utility of the SPT in everyday practice.

Analysis of patients not allocated to treatment and those withdrawn from the study provides some insight into optimal patient selection and the challenges faced with each therapy. No significant difference between completers and dropouts was found in baseline characteristics (Table 1). Most dropouts in the OAT group were seen before the start of treatment. Dentition played an important role in the initiation to treatment, and prevented device use in 33% of OAT patients who did not start the allocated treatment. It has been reported that dental limitation might preclude the use of OAT in up to 34% of all OSA cases [29]. In our study, the rate of adverse events over the first year of therapy was higher in the OAT group than in the SPT group. However, more subjects discontinued use of the SPT due to AEs between the 3- and 12-month assessments (14 vs. 6 for OAT), although the number of completers was the same in both groups (*n* = 29). Within the non-completers, the rate of persistent AHI was similar between groups as reason for dropout. Tooth pain was mentioned in the OAT group, while in the SPT group persistent snoring, joint problems, nasal problems, and broken ribs were reported as reason for dropout. Due to the mechanism of action of the SPT device, AHI and continuous snoring in the lateral position are not decreased. Just as dentition may play a role in patient selection for OAT, high lateral AHI and/or lateral snoring may be factors that identify patients less suitable for the SPT. Knowledge of the advantages, disadvantages, and adverse effects with each therapy can help guide clinicians to proper individual therapy selection and follow-up regimes that maximize adherence and long-term outcomes.

### Study limitations

The main limitation of this study was the slightly higher than expected observed dropout rate at 3 months. We mitigated this by performing additional ITT analyses on the primary outcome, using the Last-Observation-Carried-Forward method. The relatively low number of patients at 12 months could also be raised as a concern; however, the 20% dropout rate was predicted for the 3-month assessment as the primary outcome, and therefore more dropout would have been expected at 12 months. Regardless, we have included a sensitivity analysis to demonstrate the robustness of our results to the high dropout rate at 3 and 12 months. The best and worst case scenarios demonstrate the maximum and minimum bounds for the treatment effects (respectively) under different missing mechanisms for the treatment and control groups. The best case scenario assumed a 50% decrease in AHI from baseline for patients with missing data in the SPT group compared to a 0% change in AHI from baseline for patients with missing data in the OAT group. The worst case scenario assumed a 0% change in AHI from baseline for patients with missing data in the SPT group compared to a 50% decrease in AHI from baseline for patients with missing data in the OAT group. The results from these analyses demonstrate the extremes that would be expected if the missingness in the SPT and OAT groups occurred for contrasting reasons (Table S3).

## Conclusion

The results of this study show that the efficacy of SPT was maintained over 12 months of therapy, and was comparable to that of OAT in patients with mild to moderate POSA. Adherence to both treatment modalities was high, and similar in the two groups. Good long-term adherence can make an important contribution to the ongoing effectiveness of treatment in clinical practice.

## Electronic supplementary material

Table S1 (DOCX 31 kb)

Table S2 (DOCX 26 kb)

Table S3 (DOCX 27 kb)
