# Correction to: Durability of treatment effects of the sleep position trainer versus oral appliance therapy in positional OSA: 12-month follow-up of a randomized controlled trial

**DOI:** 10.1007/s11325-017-1580-8

**Published:** 2017-10-26

**Authors:** Maurits H. T. de Ruiter, Linda B. L. Benoist, Nico de Vries, Jan de Lange

**Affiliations:** 1 Department of Oral and Maxillofacial Surgery, Academic Medical Center, Amsterdam, The Netherlands; 2 Department of Otorhinolaryngology Head and Neck Surgery, OLVG West, Jan Tooropstraat 164 1061 AE, Amsterdam, The Netherlands; 3 Departments of Otorhinolaryngology and Head and Neck Surgery, Erasmus University Medical Center, Rotterdam, The Netherlands; 4 Department of Oral Kinesiology of the Academic Centre for Dentistry Amsterdam (ACTA), Amsterdam, The Netherlands; 5 Departments of Otolaryngology and Head and Neck Surgery, Antwerp University Hospital, Antwerp, Belgium

**Correction to: Sleep Breath**

10.1007/s11325-017-1568-4

The article “Durability of treatment effects of the Sleep Position Trainer versus oral appliance therapy in positional OSA: 12-month follow-up of a randomized controlled trial”, written by Maurits H. T. de Ruiter, Linda B. L. Benoist, Nico de Vries, and Jan de Lange, was originally published electronically on the publisher’s internet portal SpringerLink on 15 September 2017 without open access.

With the authors’ decision to opt for Open Choice the copyright of the article changed on October 2017 to © The Author(s) 2017 and the article is forthwith distributed under the terms of the Creative Commons Attribution 4.0 International License (http://creativecommons.org/licenses/by/4.0/), which permits use, duplication, adaptation, distribution and reproduction in any medium or format, as long as you give appropriate credit to the original author(s) and the source, provide a link to the Creative Commons license, and indicate if changes were made.

The original article was corrected.

