# Supplementary material for: Durability of treatment effects of the Sleep Position Trainer versus oral appliance therapy in positional OSA: 12-month follow-up of a randomized controlled trial
Source: Sleep Breath. 2017 Sep 15;22(2):441–50. doi: 10.1007/s11325-017-1568-4 (PMC5918490; doi:10.1007/s11325-017-1568-4)
Supplement: Supplementary file 1 — (DOCX 31 kb) [file 11325_2017_1568_MOESM1_ESM.docx]

**Table S1.** Primary and secondary outcome variables (Intention-to-treat analysis)

|  | **SPT (n=48)** | | | **OAT (n=51)** | | |
| --- | --- | --- | --- | --- | --- | --- |
|  | **Baseline** | **3 months** | **12 months** | **Baseline** | **3 months** | **12 months** |
| **Primary outcome** |  |  |  |  |  |  |
| Total AHI, /h | 13.0 (9.7,18.5) | **7.0 (3.8,12.8)^a^** | **8.0 (5.1,12.9)^a^** | 11.7 (9.0,16.2) | **9.1 (4.9,11.7)^a^** | **8.5 (4.8,11.7)^a^** |
| ODI, /h | 10.5 (7.0,15.8) | **5.5 (3.0,10.0)^a^** | **7.0 (4.0,13.0)^a^** | 9.0 (6.0,14.0) | **7.0 (4.0,10.0)^b^** | 7.0 (4.0,11.0) |
| **Secondary outcomes** |  |  |  |  |  |  |
| Supine AHI, /h | 27.0 (18.7,43.0) | **12.4 (0.4,32.8)^a^** | **14.1 (1.8,30.0)^a^** | 25.8 (17.4,35.0) | **18.9 (9.1,27.0)^a^** | **16.8 (7.6,26.6)^a^** |
| Non-supine AHI, /h | 3.5 (1.6,5.7) | 4.4 (1.8,8.5) | 3.5 (1.7,6.3) | 3.1 (1.0,5.0) | 1.9 (0.9,4.1) | 2.5 (0.7,4.1) |
| Supine sleep, % | 44.5 (30.0,55.5) | **11.5 (1.3,24.5)^a,e^** | **22.5 (5.0,42.8)^a,e^** | 41.5±18.8 | 40.9±23.0 | 42.4±20.4 |
| AI, /h | 9.0 (5.0,15.0) | **4.0 (1.0,9.3)^a^** | **4.0 (2.0,7.8)^a^** | 8.0 (4.0,12.0) | **3.0 (1.0,8.0)^a^** | **4.0 (2.0,8.0)^a^** |
| Sleep efficiency, % | 92.0 (84.5,95.0) | 91.0 (86.0,95.0) | 91.0 (86.0,95.0) | 92.0 (86.0,95.0) | 92.0 (85.0,96.0) | 93.0 (87.0,96.0) |
| Average SpO_2_, % | 95.0 (94.0,96.8) | 96.0 (95.0,96.8) | 95.5 (94.0,97.0) | 95.0 (94.0,96.0) | 95.0 (94.0,96.0) | 95.0 (94.0,96.0) |
| SBP, mmHg | 133.5 (125.0,150.0) | **130.0 (120.0,140.0)^b,g^** | 130.0 (120.0,150.0) | 130.0 (120.0,140.0) | 125.0 (120.0,135.0) | 128.0 (120.0,138.0) |
| DBP, mmHg | 90.0 (80.0,97.5) | **85.0 (75.0,90.0)^b^** | **82.5 (80.0,97.3)^c^** | 85.0 (80.0,90.0) | 85.0 (80.0,90.0) | 80.0 (80.0,86.0) |
| Heart rate, bpm | 69.0 (64.0,78.0) | 72.0 (64.3,80.0) | 72.0 (65.0,80.0) | 72.0 (66.0,80.0) | 72.0 (67.0,80.0) | 72.0 (67.0,82.0) |
| ESS score* | 7.5 (4.0,12.0) | 7.0 (5.0,10.0) | 6.0 (3.8,10.0) | 8.0 (4.0,13.0) | 7.0 (3.0,12.0) | 8.0 (3.0,12.5) |
| FOSQ score** | 19.0 (17.3,19.7) | 19.3 (16.9,19.8) | 19.3 (17.2,19.7) | 18.4 (16.2,19.7) | 18.3 (16.2,19.5) | 18.3 (16.3,19.6) |

Values are mean ± standard deviation or median (interquartile range)

*AHI* apnea-hypopnea index, *AI* apnea index, *bpm* beats/min, *DBP* diastolic blood pressure, *ESS* Epworth Sleepiness Scale, *FOSQ* Functional Outcomes of Sleep Questionnaire, *OAT* oral appliance therapy, *ODI* oxygen desaturation index, *SBP* systolic blood pressure, *SpO_2_* oxygen saturation, *SPT* Sleep Position Trainer

Missing data during follow-up coded as no-change.

*Data available in 42 patients in the SPT group and 45 patients in the OAT group

**Data available in 33 patients in the SPT group and 40 patients in the OAT group

P-values are adjusted for multiple comparisons by a Bonferroni correction. ^a^p<0.001 vs baseline (Wilcoxin signed rank test); ^b^p<0.01 vs baseline (Wilcoxin signed rank test); ^c^p<0.05 vs baseline (Wilcoxin signed rank test); ^d^p<0.05 for 12-month vs 3-month value; ^e^p<0.001 vs OAT (Mann-Whitney U test); ^f^p<0.01 vs OAT (Mann-Whitney U test); ^g^p<0.05 vs OAT (Mann-Whitney U test)
