# Supplementary material for: Durability of treatment effects of the Sleep Position Trainer versus oral appliance therapy in positional OSA: 12-month follow-up of a randomized controlled trial
Source: Sleep Breath. 2017 Sep 15;22(2):441–50. doi: 10.1007/s11325-017-1568-4 (PMC5918490; doi:10.1007/s11325-017-1568-4)
Supplement: Supplementary file 2 — (DOCX 26 kb) [file 11325_2017_1568_MOESM2_ESM.docx]

**Table S2.** Objective adherence and device usage (Intention-to-treat analysis)

|  | **SPT (n=48)** | **OAT (n=51)** | **P-value^a^** |
| --- | --- | --- | --- |
| Total nights | 252.7±139.5 | 195.5±158.2 | 0.059 |
| Total nights with adherence >4h | 163.5±121.8 | 143.1±135.9 | 0.433 |
| Average hours of use per night | 3.1±3.1 | 2.7±2.9 | 0.522 |
| Adherence >4h on 7 days in a week,  % patients | 57.4±34.6 | 47.8±40.9 | 0.208 |
| Adherence >4h on 5 days in a week,  % patients | 68.9±37.7 | 54.5±44.5 | 0.086 |

Values are mean ± standard deviation

*OAT* oral appliance therapy, *SPT* Sleep Position Trainer

^a^Independent T-test
