# Supplementary material for: Durability of treatment effects of the Sleep Position Trainer versus oral appliance therapy in positional OSA: 12-month follow-up of a randomized controlled trial
Source: Sleep Breath. 2017 Sep 15;22(2):441–50. doi: 10.1007/s11325-017-1568-4 (PMC5918490; doi:10.1007/s11325-017-1568-4)
Supplement: Supplementary file 3 — (DOCX 27 kb) [file 11325_2017_1568_MOESM3_ESM.docx]

**Table S3.** Sensitivity Analysis (Intention-to-treat analysis)

|  | **SPT (n=48)** | | | | **OAT (n=51)** | | | |
| --- | --- | --- | --- | --- | --- | --- | --- | --- |
|  | **Baseline** | **∆ Baseline to 3 months** | **∆ Baseline to 12 months** | **∆ 3 to 12 months** | **Baseline** | **∆ Baseline to 3 months** | **∆ Baseline to 12 months** | **∆ 3 to 12 months** |
| **Best case scenario** |  |  |  |  |  |  |  |  |
| Total AHI, /h | 13.0 (9.7,18.5) | **-5.5 (-9.7,-1.5)^a^** | **-6.4 (-10.4,-3.1)^a,e^** | **-3.0 (-6.9,1.7)^b^** | 11.7 (9.0,16.2) | **-2.8 (-7.3,0.0)^a^** | **0.0 (-7.9,0.0)^a^** | 0.0 (-1.1,0.7) |
| **Worst case scenario** |  |  |  |  |  |  |  |  |
| Total AHI, /h | 13.0 (9.7,18.5) | **-5.2 (-9.7,-1.1)^a^** | **-1.0 (-8.8,0.0)^a^** | **0.0 (-0.9,1.7)^e^** | 11.7 (9.0,16.2) | **-5.2 (-7.6,-3.2)^a^** | **-5.5 (-9.2,-3.3)^a^** | **-4.3 (-5.9,0.7)^a^** |

Values are mean ± standard deviation or median (interquartile range)

∆ change, *AHI* apnea-hypopnea index, *OAT* oral appliance therapy, *SPT* Sleep Position Trainer

P-values are adjusted for multiple comparisons by a Bonferroni correction. ^a^p<0.001 vs baseline (Wilcoxin signed rank test); ^b^p<0.01 vs baseline (Wilcoxin signed rank test); ^c^p<0.05 vs baseline (Wilcoxin signed rank test); ^d^p<0.05 for 12-month vs 3-month value; ^e^p<0.001 vs OAT (Mann-Whitney U test); ^f^p<0.01 vs OAT (Mann-Whitney U test); ^g^p<0.05 vs OAT (Mann-Whitney U test)
